# Association between periodontitis and peripheral artery disease: a systematic review and meta-analysis

**DOI:** 10.1186/s12872-018-0879-0

**Published:** 2018-07-06

**Authors:** Shuo Yang, Li Sheng Zhao, Chuan Cai, Quan Shi, Ning Wen, Juan Xu

**Affiliations:** Department of Stomatology, Chinese People’s Liberation Army General Hospital, 28 Fuxing Road, Beijing, 100853 China

**Keywords:** Periodontitis, Peripheral arterial disease, Inflammation, Risk factor, Meta-analysis

## Abstract

**Background:**

Inflammation is a common feature of both peripheral arterial disease (PAD) and periodontitis. Some studies have evaluated the association between PAD and periodontitis. However, there is still no specialized meta-analysis that has quantitatively assessed the strength of the association. Thus, we conducted this meta-analysis to critically assess the strength of the association between PAD and periodontitis.

**Methods:**

PubMed, Embase, and the Cochrane Library were searched for observational studies of the association between periodontitis and PAD in February 2018. Risk ratios (RRs) and their 95% confidence intervals (CIs) from included studies were pooled to evaluate the strength of the association between periodontitis and PAD. Weighted mean differences (WMDs) and their 95% CIs were pooled to compare the difference in periodontal-related parameters between PAD and non-PAD patients.

**Results:**

Seven studies including a total of 4307 participants were included in the meta-analysis. The pooled analysis showed that there was a significant difference in the risk of periodontitis between PAD patients and non-PAD participants (RR = 1.70, 95% CI = 1.25–2.29, *P* = 0.01). There was also a significant difference in number of missing teeth between PAD patients and non-PAD participants (WMD = 3.75, 95% CI = 1.31–6.19, *P* = 0.003). No significant difference was found in clinical attachment loss between PAD patients and non-PAD participants (WMD = − 0.05, 95% CI = − 0.03–0.19, *P* = 0.686).

**Conclusion:**

In conclusion, the results of this meta-analysis revealed a significant relationship between periodontitis and PAD. Moreover, our study indicated that PAD patients had more missing teeth than control subjects did. Further high-quality and well-designed studies with specific inclusion and exclusion criteria are required to strengthen the conclusions of this study.

**Electronic supplementary material:**

The online version of this article (10.1186/s12872-018-0879-0) contains supplementary material, which is available to authorized users.

## Background

Periodontitis, a chronic inflammatory disease, is primarily characterized by the destruction of tooth-supporting tissues [1, 2]. Without treatment, periodontitis typically causes a loss of connective tissue attachment, erosion of the alveolar bone and, ultimately, tooth loss [3]. In the US, nearly half of the population aged > 30 years have periodontal problems, and nearly 10% of them have severe periodontitis [4]. The findings from current studies indicate that periodontitis is associated with a wide range of systemic diseases, including pulmonary disease, diabetes mellitus, myocardial infarction, rheumatoid arthritis, and systemic lupus erythematosus [5–8]. The World Health Organization (WHO) has stated that oral health, including periodontal health, is an essential part of general health [9].

Higher levels of serum IL-6, C-reactive protein (CRP), TNF-α and IL-1β have been reported among periodontitis patients in several studies [10, 11]. Additionally, the serum IL-6 and CRP levels have a positive association with the extent of periodontitis [12]. These results suggest that as a chronic inflammatory condition, periodontitis may contribute to increased serum inflammatory markers.

Peripheral artery disease (PAD), which is usually associated with atherosclerosis, results in a significant reduction of the lumen of peripheral arteries, and its most common symptom is intermittent claudication [13]. Studies have shown that PAD is associated with elevated morbidity and mortality with cardiovascular disease (CVD) [14, 15]. The presence of PAD leads to a three- to six-fold increase in the risk of CVD mortality [16]. PAD shares the same underlying pathology as CVD and cerebrovascular diseases [17, 18]. Systemic hyperinflammation plays an important role in the onset of these diseases. It has been reported that elevated circulating levels of IL-6, TNF-α and CRP are associated with the progression of CVD, PAD and cerebrovascular disease [19–21].

The relationship between periodontitis and CVD has been investigated in many studies, and there is strong evidence that periodontitis is associated with CVD [22–24], as PAD and CVD are both chronic infammatory conditions, and they share similar infammatory factors with periodontitis. We hypothesized that there may also exist an association between PAD and periodontitis. Mendez et al. [25] was the first to report that subjects with clinically significant periodontitis at baseline had a 2.27-fold possibility of developing PAD (OR = 2.27, 95% CI = 1.32–3.9). In a case–control study conducted by Soto-Barreras et al. [26], periodontitis, defined as a clinical attachment loss (CAL) ≥4 mm in at least 30% of the six measured sites, was strongly associated with PAD risk (OR = 8.18, 95% CI = 1.21–35.23). Similar findings have proliferated in recent years [27, 28]. However, no specialized meta-analysis has quantitatively assessed the strength of the association between PAD and periodontitis. Thus, we conducted this meta-analysis to evaluate the possible association between PAD and periodontitis. The results of our study will expand the current knowledge of the etiology of PAD and will provide clinicians with better evidence-based recommendations and management strategies.

## Methods

We conducted this meta-analysis in accordance with the Preferred Reporting Items for Systematic Reviews and Meta-Analyses statement (PRISMA) [29]. The checklist of the PRISMA guidelines has been put into the supplemental material (Additional file 1). This study was conducted according to the Population, Intervention, Control and Outcome (PICO) format, in order to answer the following focused PICO question:

- Are people (P) with periodontitis (I) more likely to get PAD (O)?
- Population: humans with or without PAD
- Intervention: participants with periodontitis
- Comparison: non-PAD participants with periodontitis
- Outcome: PAD

### Literature-search strategy

We performed a literature search in February 2018, and the search language was restricted to English. PubMed, Embase, and the Cochrane Library were searched using the following key words: “periodontitis,” “periodontal disease,” “peripheral vascular disease,” and “peripheral arterial disease.” In addition, we identified additional studies by checking the reference lists of the related studies. The search strategy (Additional file 2) has been put into the supplemental material.

### Inclusion and exclusion criteria

We included studies that (1) reported the relationship between periodontitis and risk of PAD; (2) defined PAD by ankle brachial pressure index (ABI) or angiographic findings or clinical symptoms [30]; (3) defined periodontitis using at least one of several clinical definitions according to the International Workshop for the Classification of Periodontal Disease [31] or by self-report using questionnaires or clinical diagnosis by a periodontist; (4) were observational, including those with a cross-sectional, case-control, or cohort design; and (5) had data that could be extracted.

The exclusion criteria were (1) animal model or in vitro studies; (2) reviews, case reports or comments; (3) non-English-language studies; and (4) studies without available data.

### Study selection methods

To select studies, we first excluded duplicated studies from the literature search. Then, we screened the titles and abstracts and excluded obviously irrelevant studies. After assessing the full texts of potentially eligible studies, we only included studies that met the inclusion criteria. The entire process was conducted by 2 reviewers, and any disagreements were resolved by discussion with a third reviewer.

### Data extraction and quality assessment

Two authors (SY and LSZ) independently assessed the characteristics of included studies. The following information was extracted from each included study: first author’s name and year of publication; country of study; study design; characteristics of the study participants, including number of patients and controls, age range, and sex; study population; definition of PAD and periodontitis; and adjusted or matched factors.

The Newcastle-Ottawa Scale (NOS) [32] was used by two authors (CC and QS) to complete the quality assessment of all eligible studies. The NOS ratings (Additional file 3) has been put into the supplemental material. In this assessment tool, study selection, comparability, and outcome were used to appraise the methodological quality of the included studies, with a maximum of 9 points for each study. NOS scores of 1–3, 4–6, and 7–9 indicated low, moderate, and high study quality, respectively.

### Statistical analysis

Statistical analyses were performed using the Stata 12.0 software (Stata Corporation, College Station, TX, USA). The risk ratios (RRs) and 95% confidence intervals (CIs) from the included studies were pooled to evaluate the strength of the association between periodontitis and PAD. As the parameters used for evaluating periodontal status were continuous variables, weighted mean differences and their 95% CIs were pooled to compare the difference in periodontal status between PAD and non-PAD patients.

The I^2^ statistic was used to assess the degree of heterogeneity among studies. The values 25, 50, and 75% corresponded to low, moderate, and high heterogeneity, respectively. A fixed effects model was applied if I^2^ < 50%, and a random effects model was used if I^2^ > 50%.

## Results

### Literature selection

**Fig. 1:**
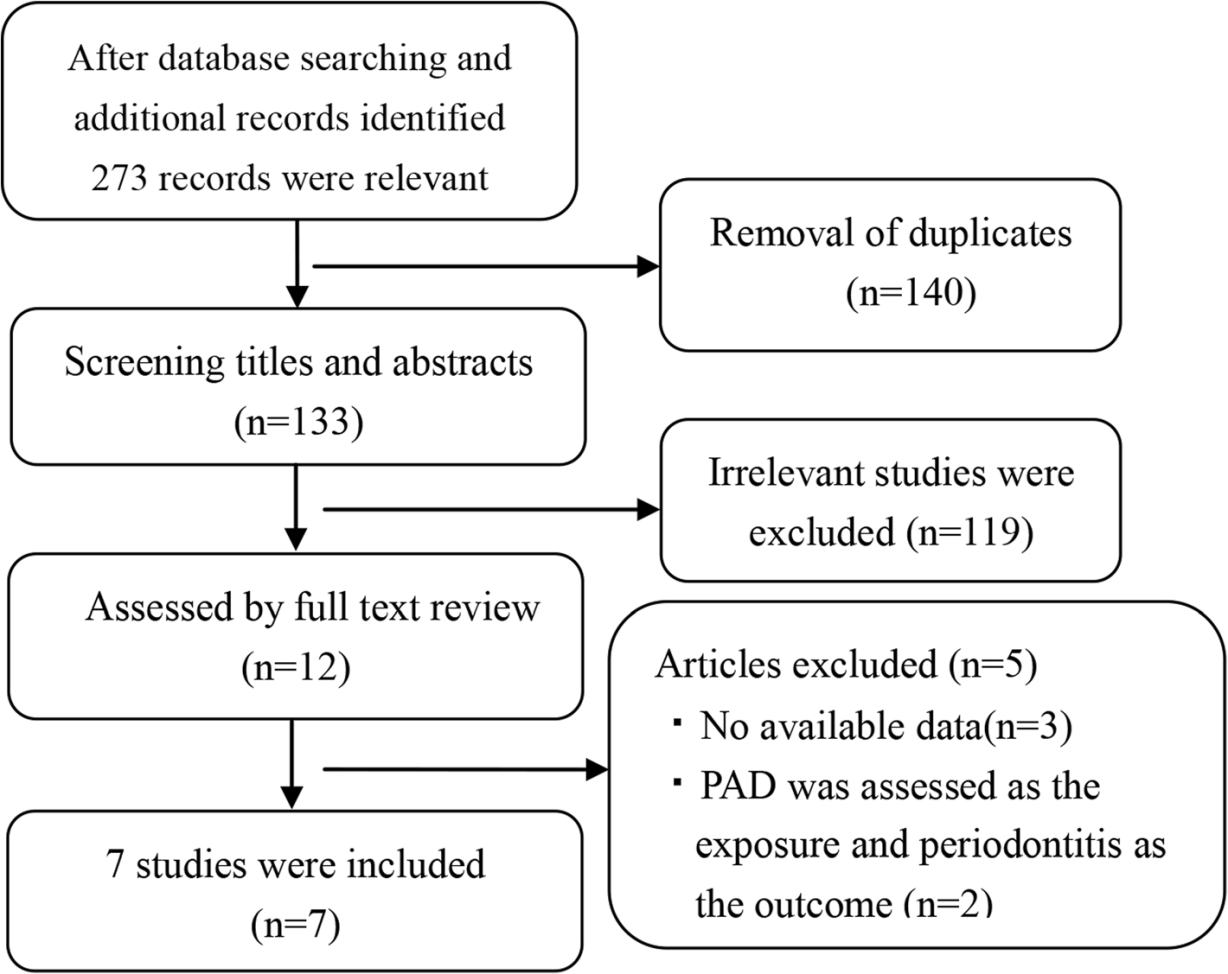
Study flow diagram

The detailed literature selection process is shown in Fig. 1. Based on the search strategy, 273 potentially relevant studies were selected from the electronic database. Of these studies, 140 were excluded after removing duplicates. After screening titles and abstracts, 119 irrelevant studies were excluded because they failed to meet the eligibility criteria. Twelve studies were subsequently assessed by full text review, and 5 studies were removed for various reasons. Ultimately, 7 studies were included in our meta-analysis [25–28, 30, 33, 34].

### Study characteristics

The characteristics of the included studies are shown in Tables 1 and 2. A total of 4307 participants were included in the meta-analysis, for a total of 493 participants with PAD and 3814 participants without PAD. The publication dates of the included studies ranged from 1998 to 2017. Of the seven included studies, four [26, 27, 30, 33] were case-control studies, two [28, 34] were cross-sectional studies, and one [25] was a prospective cohort study. Three studies [27, 28, 30] were conducted in Asian countries, two studies [25, 26] were conducted in American countries, and two [33, 34] were conducted in European countries. One study [25] enrolled male participants only, and one [33] enrolled female participants only.

**Table 1:** Characteristics of included studies

Study (Author, Year)	Country	Study population	Study Design	PAD^a^ patients		Control	
				Number (M/F)^b^	Age (SD^c^ or Range)	Number (M/F)	Age (SD or Range)
Aoyama et al. 2017 [22]	Japan	hospital-based	cross-sectional	34 (23/11)	65.6 ± 11.8	956 (693/263)	64.4 ± 13.0
Çalapkorur et al. 2017 [34]	Turkey	hospital-based	cross-sectional	40 (32/8)	60.45 ± 9.94	20 (18/2)	57.40 ± 11.16
Ahn et al. 2016 [27]	South Korea	population-based	case-control	72 (28/44)	NR^d^	1271 (473/798)	NR
Soto-Barreras et al. 2013 [26]	Mexico	hospital-based	case-control	30 (8/22)	61.86 ± 8.49	30 (9:21)	63.23 ± 9.06
Chen et al. 2008 [30]	Japan	hospital-based	case-control	25 (21/4)	67.6 ± 10	32 (28/4)	63.1 ± 10
Bloemenkamp et al. 2002 [33]	Netherlands	population-based	case-control	212 (0/212)	48.2 ± 7.0	475 (0/475)	45.5 ± 8.1
Mendez et al. 1998 [25]	USA	population-based	cohort	80 (80/0)	44.2 (29–62)	1030 (1030/0)	42.7 (23–80)

^a^PAD, peripheral arterial disease

^b^M/F, male/female

^c^SD, standard deviation

^d^NR, not report

**Table 2:** Characteristics of included studies

Study (Author, Year)	Defnition of PAD	Defnition of periodontitis	Adjusted or matched factors	NOS score
Aoyama et al. 2017 [22]	PAD was diagnosed based on clinical symptoms, ABI, and angiographic fndings	NR	age, sex, smoking, hypertension, dyslipidemia and HbA1c levels	6
Çalapkorur et al. 2017 [34]	Patients with ABI values of ≤0.90 were diagnosed as having PAD	Periodontitis was defined as the presence of at least five teeth with one or more sites with a PD of ≥5 mm, a CAL of ≥2 mm, the presence of BOP and 30% radiographic bone loss	age, gender, diabetes, hypertension and BMI	6
Ahn et al. 2016 [27]	PAD was evaluated by using ABI lower than 1.0	The radiographic alveolar bone loss 4 mm at two or more interproximal sites, not on the same tooth	age, sex, education level, tooth loss, smoking, drinking, central obesity	7
Soto-Barreras et al. 2013 [26]	Patients with ABI values of ≤0.90 were diagnosed as having PAD	The diagnosis of periodontitis was determined when the attachment loss was ≥4 mm in ≥30% of measured sites.	age, sex, BMI,smoking, and diabetes mellitus	8
Chen et al. 2008 [30]	PAD was diagnosed based on clinical symptoms, ABI, and angiographic fndings	Participants who presented with at least one probing site with PD 4 mm or CAL 4 mm in each quadrant were defined as periodontitis patients	Smoking, age, gender, and diabetes	7
Bloemenkamp et al. 2002 [33]	PAD was angiographically confirmed when a stenotic lesion causing more than 50% reduction of the lumen was present in at least one major peripheral artery	NR	Smoking, age, gender, and diabetes	7
Mendez et al. 1998 [25]	PAD was defined as one or more of the following: (1) intermittent claudication; (2) extracranial erebrovascular disease; (3) atherosclerosis (including aortic, renal, and mesenteric disease); and (4) arterial embolism and thrombosis.	Periodontitis was considered present if the mean whole mouth alveolar bone loss was>20%.	age, BMI, family history of heart disease, and smoking exposure	8

*ABI* ankle brachial pressure index, *PAD* peripheral arterial disease, *PD* probing depth, *CAL* clinical attachment loss, *BOP* bleeding on probing, *BMI* body mass index, *NR* not report

The quality of the included studies was assessed using the NOS; the results are shown in Table 2. Four case-control studies [26, 27, 30, 33] and one cohort study [25] scored more than 6 points and were considered to be of high quality. Two cross-sectional studies [28, 34] scored 6 points and were considered to be of moderate quality.

### Meta-analysis

#### PAD and periodontitis prevalence

**Fig. 2:**
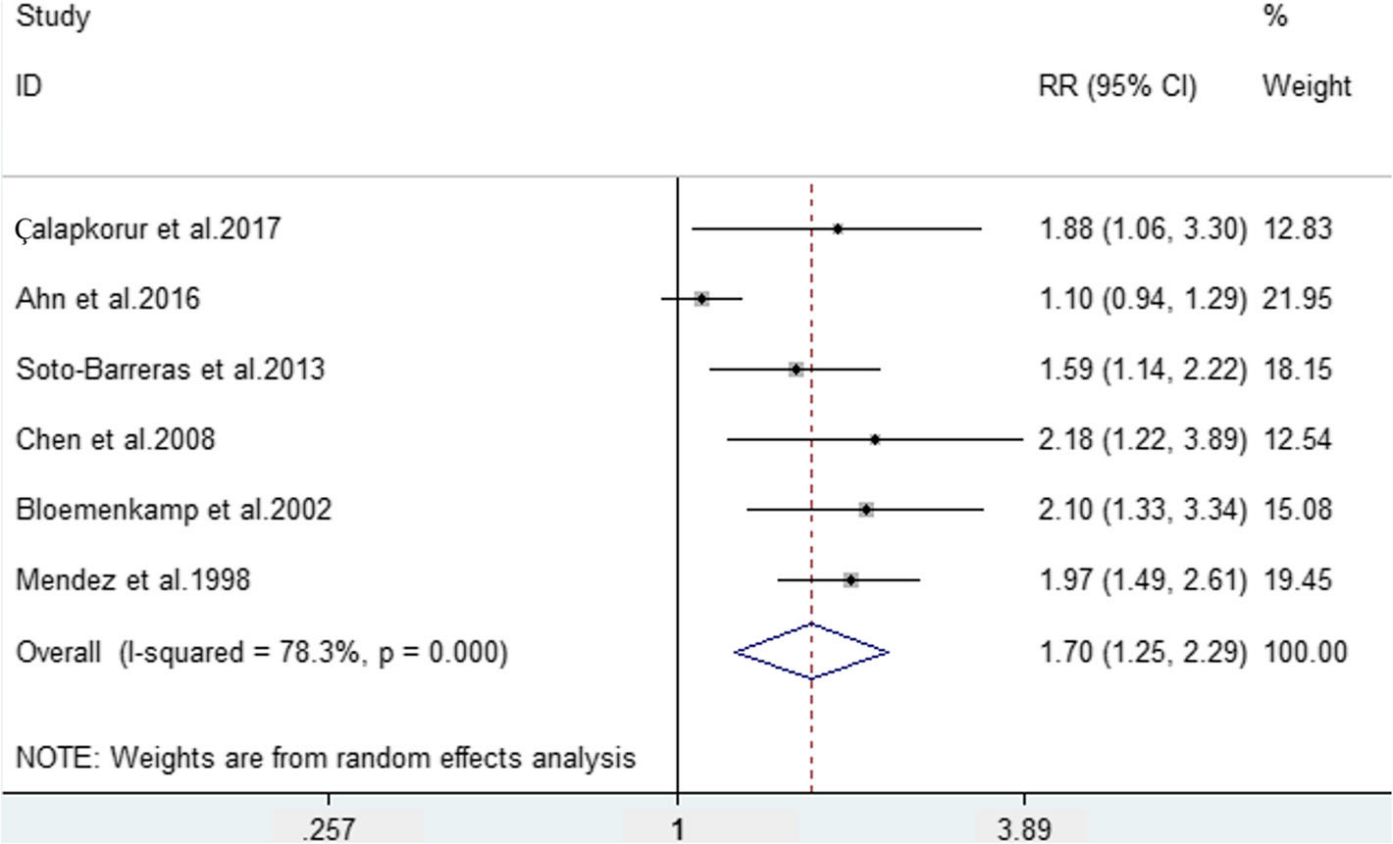
Forest plot of the risk of periodontitis between PAD patients and non-PAD participants

Six studies [25–27, 30, 33, 34] that had available data to calculate the RRs and 95% CIs were included in the meta-analysis to evaluate the strength of the association between PAD and periodontitis. The pooled analysis indicated a significant difference in the risk of periodontitis between PAD patients and non-PAD participants (RR = 1.70, 95% CI = 1.25–2.29, *P* = 0.01, Fig. 2). However, considering the high heterogeneity (I^2^ = 78.3%) of the included studies, a random effects model was used.

#### Missing teeth

**Fig. 3:**
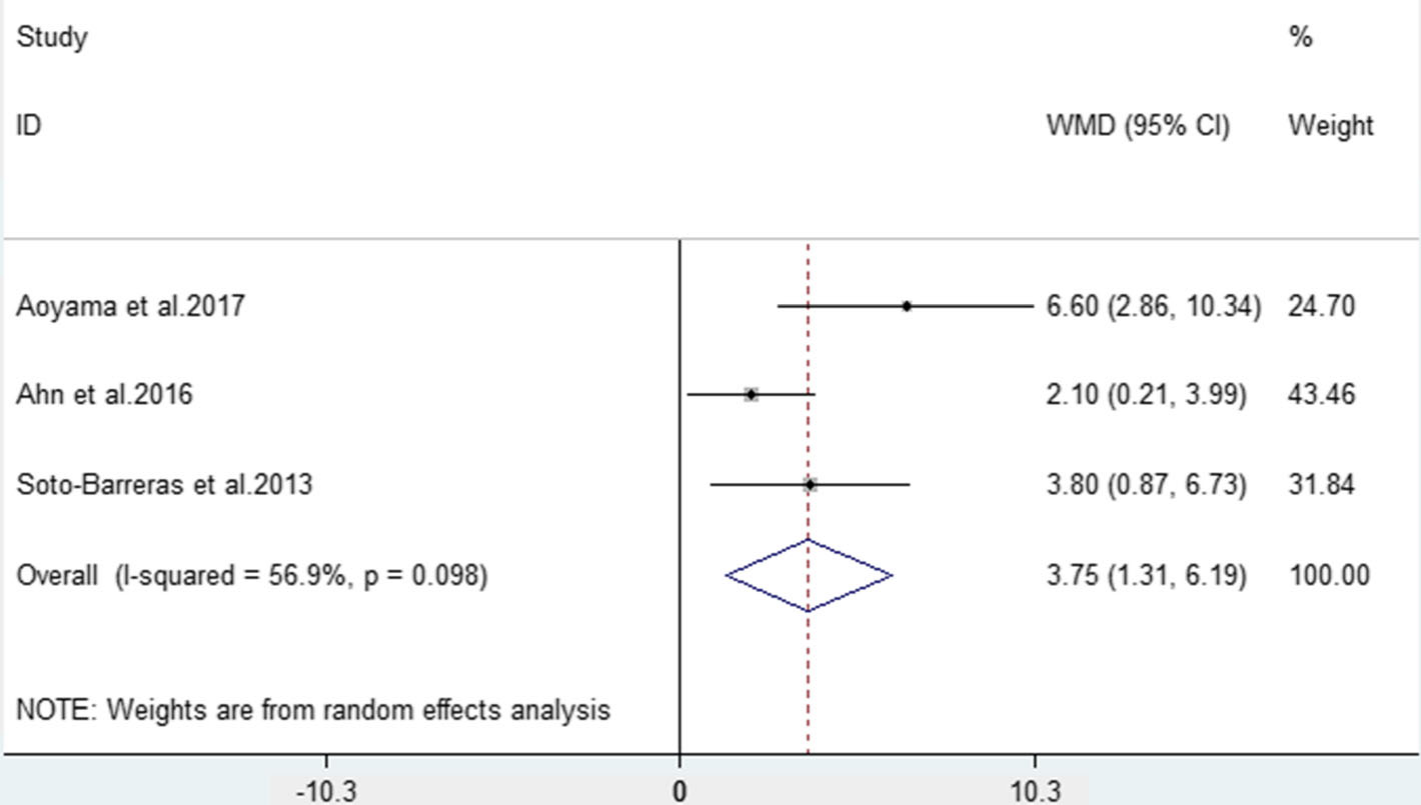
Forest plot of the weighted mean difference in missing teeth between PAD patients and non-PAD participants

Three studies [26–28] compared the number of missing teeth in PAD patients and non-PAD participants. The meta-analysis showed that PAD patients lost more teeth than non-PAD participants did, and the difference was statistically significant (WMD = 3.75, 95% CI = 1.31–6.19, *P* = 0.003, Fig. 3). Considering the high heterogeneity (I^2^ = 56.9%) of the included studies, a random effects models was used.

#### Clinical attachment loss (CAL)

**Fig. 4:**
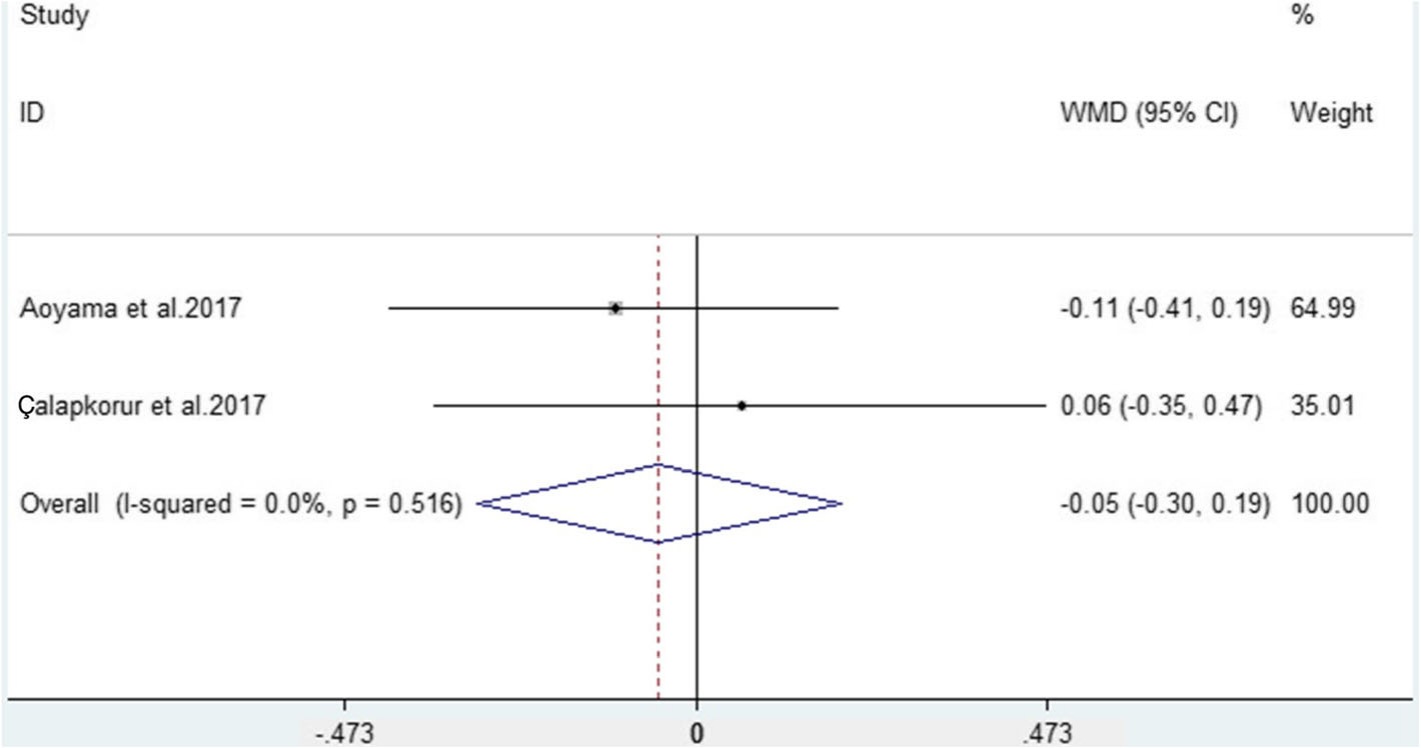
Forest plot of the weighted mean difference in CAL between PAD patients and non-PAD participants

Two studies [28, 34] reported CAL results in PAD patients and non-PAD participants. The pooled analysis showed that there was no significant difference in clinical attachment loss between PAD patients and non-PAD participants (WMD = − 0.05, 95% CI = − 0.03–0.19, *P* = 0.686, Fig. 4). Considering the low heterogeneity (I^2^ = 0.0%) of the included studies, a fixed effects model was used.

## Discussion

In this meta-analysis, we found a statistically significant increased risk of periodontitis in PAD patients compared to non-PAD participants, suggesting that there was a significant association between PAD and periodontitis. Moreover, we found that PAD patients had more missing teeth than non-PAD participants did. However, there was no statistical difference in CAL between PAD patients and controls.

Our finding that periodontitis was associated with PAD is in agreement with other studies focused on this relationship. Ahn et al. [27] reported that periodontitis was a risk factor for PAD; their study showed that patients with periodontitis had a 2.03-fold increase in the risk of PAD (95% CI = 1.05–3.93). Çalapkorur et al. [34] showed that periodontitis raised the odds ratio for developing PAD to 5.84 (95% CI = 1.56–21.91). Chen et al. [30] conducted a case-control study and found that periodontitis was associated with a relative risk of 5.45 (95% CI = 1.57–18.89) for developing PAD.

In this meta-analysis, we also compared periodontal parameters, such as the CAL and number of missing teeth, to reflect the periodontal status of PAD patients and non-PAD participants. CAL is well known as a gold-standard measurement for periodontitis in both clinical research and clinical work [35]. However, this meta-analysis failed to find a difference in CAL between PAD patients and non-PAD participants. This null finding may have been caused by the heterogeneity of the study groups and the limited number of related studies. Hence, further well-designed studies are needed to evaluate this relationship.

Missing teeth can reflect an irreversible condition in the end-stage of periodontitis [36]. Our meta-analysis indicated that PAD patients typically have more missing teeth than non-PAD participants do. It is well known that the most important cause of missing teeth is periodontitis. This result, in turn, may illustrate the close relationship between periodontitis and PAD.

The mechanism by which periodontitis causes PAD is not yet fully understood. However, elevated levels of inflammatory mediators such as IL-6, IL-1β and TNF-α in systemic circulation within PAD patients suggests that chronic infection in the body that may play an important role [11, 30, 37]. Periodontitis is a chronic inflammatory disease, and it is believed that periodontitis has the ability to induce local and host immune responses and to cause both transient bacteremia and the release of inflammatory mediators such as ILs and TNF-α. These mediators can subsequently damage endothelial tissues and eventually lead to PAD [11, 38].

However, we observed high heterogeneity among the studies included in the meta-analysis. The observed heterogeneity may have been due to several factors. First, diagnostic criteria and methods of evaluating periodontitis were different among the included studies, and in some studies, the methods were not reported. These different criteria and evaluation methods for periodontitis lead to variation in the outcome measures and thus cause heterogeneity. Second, different types of observational studies and study populations were included in this study. We included cross-sectional, case-control, and cohort studies in this meta-analysis, and the hospital-based or population-based subjects were recruited in different studies. Third, different adjustments for confounding factors may play a role in heterogeneity. Some factors can affect both PAD and periodontitis independently, such as diabetes, tobacco and age. Moreover, unmeasured confounders may exist and could lead to heterogeneity. Fourth, the limited patient numbers in some studies may have contributed to the inconsistent results and resulted in high heterogeneity.

To our knowledge, this is the first meta-analysis to estimate the association between periodontitis and PAD. Our results showed a significant relationship between periodontitis and PAD. Additionally, our study indicated that PAD patients had more missing teeth than control subjects did. Furthermore, a study conducted by Blum et al. [39] found that treating periodontitis effectively improved the function of the vascular endothelium and may play a protective role in vascular injury and prevent PAD. From the perspective of public health, periodontitis is a disease that can be prevented and treated; thus, the effective implementation of prevention programs and treatment measures could not only improve oral health but may also reduce the risk of PAD. It should be noted that the current evidence only reveals a potential association of periodontitis and PAD. The specific mechanisms by which the two diseases are associated remain unknown, and further experimental studies to investigate pathologic mechanisms underlying periodontitis and PAD are needed.

The current meta-analysis has some limitations. First, high heterogeneity among the included studies was detected during analysis. Second, sensitivity analysis, meta-regression, and publication bias analyses were not performed due to the limited number of included studies. Third, the included studies were published in English; thus, relevant studies published in other languages may have been overlooked, causing selection bias.

## Conclusions

In conclusion, the results of this meta-analysis revealed a significant relationship between periodontitis and PAD. Moreover, our study demonstrated that PAD patients had more missing teeth than control subjects did. However, the results should be viewed with caution because of the high heterogeneity and limited number of included studies. Further high-quality and well-designed studies with specific inclusion and exclusion criteria are required to strengthen the conclusions of this study.

## Additional files

Additional file 1: The checklist of PRISMA guidelines. (DOC 64 kb)

Additional file 2: **Table S1.** Search strategy. (PDF 11 kb)

Additional file 3: NOS ratings. (PDF 17 kb)

## Abbreviations

ABI: Ankle brachial pressure index
CAL: Clinical attachment loss
CIs: Confidence intervals
CRP: C-reactive protein
CVD: Cardiovascular disease
NOS: Newcastle-Ottawa Scale
PAD: Peripheral arterial disease
PRISMA: Preferred Reporting Items for Systematic Reviews and Meta-Analyses statement
RRs: Risk ratios
WHO: World health organization
WMDs: Weighted mean differences
