# Supplementary material for: Association between periodontitis and peripheral artery disease: a systematic review and meta-analysis
Source: BMC Cardiovasc Disord. 2018 Jul 6;18:141. doi: 10.1186/s12872-018-0879-0 (PMC6035462; doi:10.1186/s12872-018-0879-0)
Supplement: Supplementary file 2 — Table S1. Search strategy. (PDF 11 kb) [file 12872_2018_879_MOESM2_ESM.pdf]

Table 1 The key words and database.

Database	Key words
Pubmed	(periodontitis OR periodontal disease) AND (peripheral vascular disease OR peripheral arterial disease)
Embase	(periodontitis OR periodontal disease) AND (peripheral vascular disease OR peripheral arterial disease)
Cochrane Library	(periodontitis OR periodontal disease) AND (peripheral vascular disease OR peripheral arterial disease)
